# Spontaneous Coronary Artery Dissection: An Elusive Disease of the Coronaries

**DOI:** 10.14797/mdcvj.1129

**Published:** 2022-06-03

**Authors:** Smitha Narayana Gowda, Swaminathan Perinkulam Sathyanarayanan, Alpesh Shah

**Affiliations:** 1 Houston Methodist DeBakey Heart & Vascular Center, Houston Methodist Hospital, Houston, Texas, US; 2 Department of Internal Medicine, University of South Dakota, Sioux Falls, South Dakota, US

**Keywords:** spontaneous coronary artery dissection, myocardial infarction, acute coronary syndrome

## Abstract

The articles in our Points to Remember column highlight important “need to know” facts about conditions that cardiovascular healthcare professionals may encounter. These may come from any medical specialty, such as nephrology or neurology. The article in this issue is provided by Swaminathan Perinkulam Sathyanarayanan, MD, Department of Internal Medicine, University of South Dakota, and Smitha Narayana Gowda, MD, and Alpesh Shah, MD, Houston Methodist DeBakey Heart & Vascular Center, Houston Methodist.

Spontaneous coronary artery dissection (SCAD) is an important cause of myocardial infarction. Diagnosis and management of SCAD can be challenging; therefore, clinicians should consider SCAD in the differential diagnosis, especially in young women presenting with acute coronary syndrome (ACS).

A 63-year-old female presented to the emergency room (ER) with severe substernal chest pain radiating to her arms and jaw. Her past medical history was significant for hyperlipidemia, hypothyroidism, and relapsing/remitting multiple sclerosis (MS) maintained on glatiramer acetate. Two weeks prior, she was treated for an MS flare with a 5-day course of methylprednisolone 1000 mg.

Electrocardiogram demonstrated dynamic ST/T wave changes (***Figure 1***). Her troponin-I peaked at 35.798 ng/mL (ref < 0.04 ng/mL). Echocardiography showed apical akinesia with a left ventricular ejection fraction of 45% to 50%. Coronary angiogram showed bifid left anterior descending coronary artery (LAD) with severe stenosis in the diagonal branch of the bifid LAD system. The other coronary arteries were without any significant stenosis (***Figure 2***). Based on the angiographic findings, a diagnosis of SCAD was made. She was managed with medical therapy: dual antiplatelets, statin, and betablocker. A computed tomography angiogram of head, neck, chest, abdomen, and pelvis was negative for features suggestive of fibromuscular dysplasia.

**Figure 1:**
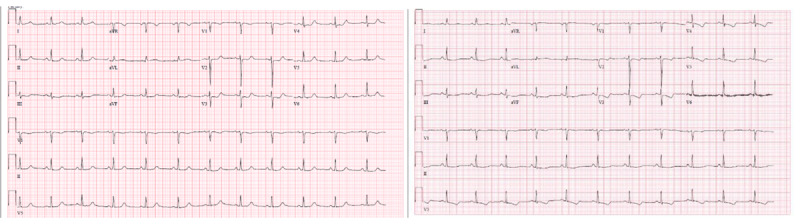
Echocardiography showing dynamic ST/T wave changes.

**Figure 2:**
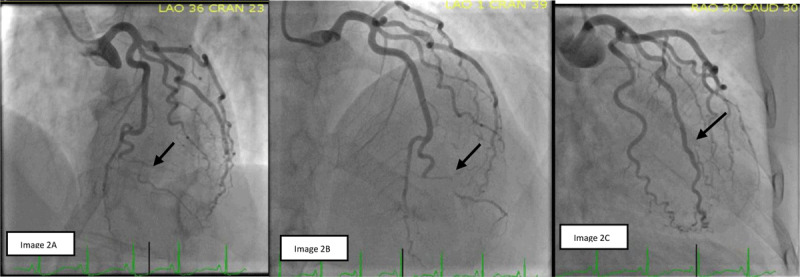
**(A, B)** Spontaneous coronary artery dissection of diagonal branch of the bifid left anterior descending coronary artery. **(C)** Normal caliber ramus coronary artery.

The patient had a similar presentation 6 months later after an MS flare treated with corticosteroids. Repeat coronary angiography showed a new area of SCAD in the obtuse marginal branch and possibly in the apical branch of the bifid LAD system. It also showed healing of the prior SCAD in the diagonal branch (***Figure 3***). She was managed conservatively with medical therapy, and her MS maintenance medication was changed to ocrelizumab, with a better response. At 6-month follow-up, she did not have any recurrence of symptoms.

**Figure 3:**
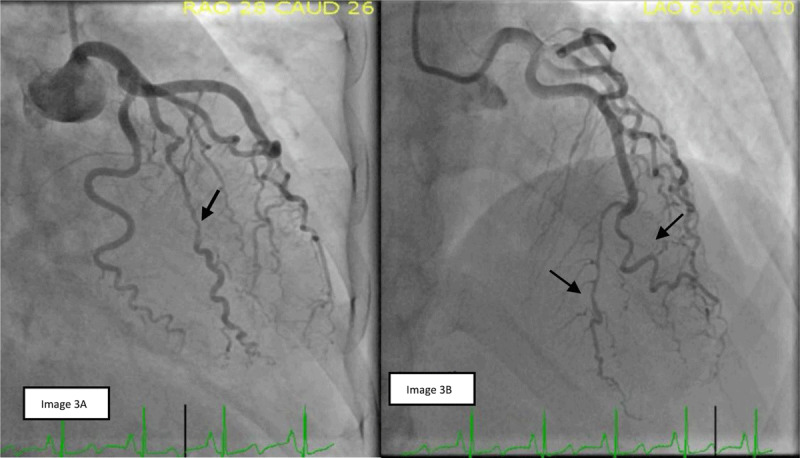
**(A)** Spontaneous coronary artery dissection (SCAD) of ramus coronary artery. **(B)** Possible SCAD in the apical branch of bifid left anterior descending coronary artery (LAD) and healing of previous SCAD in diagonal branch of bifid LAD.

## Points to Remember

**Figure 4:**
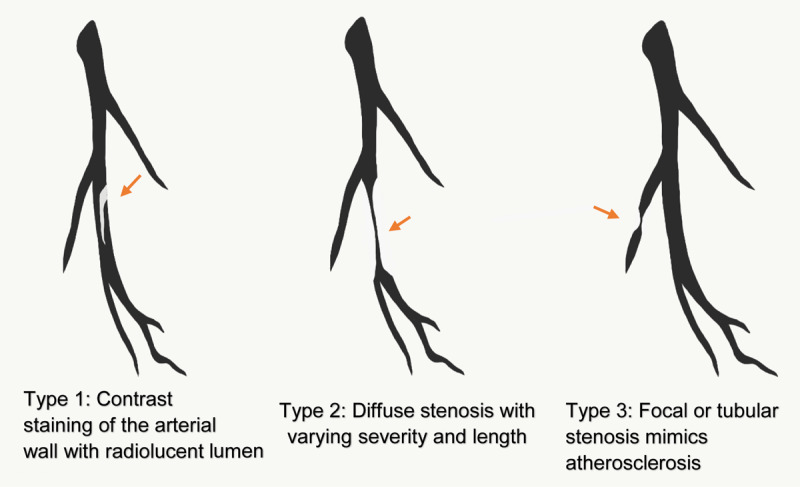
Diagnosis of spontaneous coronary artery dissection is based on coronary angiography, with appearance classified into three Yip-Saw types.

- SCAD is defined as noniatrogenic epicardial coronary artery dissection that is not associated with trauma or atherosclerosis.^1^
- The mechanism is thought to be development of intimal tear or intramural hematoma formation within the tunica media that causes compression of the true lumen of the coronary artery, leading to myocardial injury.^2^
- Between 1% and 4% of ACS cases are from SCAD. An important cause of myocardial infarction (MI) in young women, it accounts for up to 35% of ACS cases in women < 50 years of age and is the most common cause of pregnancy-associated MI.^1^
- Risk factors include female gender, pregnancy, fibromuscular dysplasia connective tissue disorders, hormonal therapies, corticosteroids, and emotional and physical stressors. Traditional risk factors of MI are generally few or absent.^3^
- Chest pain, the most common presentation of SCAD, rarely presents with cardiogenic shock, ventricular arrythmias, and sudden cardiac death.^2^
- Diagnosis of SCAD is generally made on coronary angiography. Based on its angiographic appearance (***Figure 4***), it has three types of Yip-Saw classification: (1) multiple radiolucent lumens or arterial wall contrast staining; (2) presence of diffuse stenosis of varying severity and length (most commonly seen); and (3) focal or tubular stenosis mimics atherosclerosis.^4^
- During diagnostic uncertainty, intracoronary imaging modalities such as optical coherence tomography (OCT) and intravascular ultrasound (IVUS) should be considered after weighing risk versus benefits because these maneuvers can cause extension of dissection, occlusion of the lumen, and iatrogenic dissection.^2^
- While optimal management is up for debate, conservative medical therapy is recommended in most cases. Healing of SCAD lesion occurs in a majority of patients (70–97%) after conservative management, but recurrence is common.^1^
- Revascularization with percutaneous coronary intervention or coronary artery bypass graft surgery should be considered in patients with symptoms of ongoing ischemia, hemodynamic instability, or left main dissection and/or involvement of the proximal part of the epicardial vessels.^1^
- Percutaneous coronary intervention (PCI) may be technically challenging due to difficulty assessing the true lumen and propagation of dissection antegrade and retrograde. PCI should be undertaken at a high experience center with IVUS or OCT guidance and with careful attention to complete coverage of the entire length of the lesion, especially the proximal origin of dissection.
- Long-term medical therapy includes aspirin and beta blockers. Beta blocker use has been associated with lower risk of recurrent SCAD. The role of antiplatelets, like clopidogrel, remains unclear but is used commonly in contemporary clinical practice.^5^
